# Vision and implementation for the first U.S. carbon ion therapy center part II: Design philosophy and clinical implementation

**DOI:** 10.1002/acm2.70866

**Published:** 2026-10-20

**Authors:** Xiaoying Liang, Chris J Beltran

**Affiliations:** ^1^ Department of Radiation Oncology Mayo Clinic Jacksonville Florida USA

## Abstract

The anticipated clinical demand for carbon ion radiotherapy (CIRT) in the United States is expected to include a substantial proportion of clinically complex disease sites that require accurate, highly conformal, and adaptive treatment. These clinical needs, together with the unique physical and biological characteristics of carbon ion beams, form the foundation of the design philosophy for the Mayo Clinic Florida (MCF) CIRT program. The program remains under active development, with clinical deployment planned for 2028. Building upon the clinical rationale and anticipated U.S. patient population discussed in Part I of this two‐part review, this Vision Article describes how MCF intends to develop an integrated CIRT treatment system. The program is guided by two overarching principles: fast, accurate, and safe beam delivery and accurate treatment planning and treatment delivery, supported by an integrated patient‐specific quality assurance framework. Key elements include high‐dose‐rate dose‐driven continuous scanning with advanced beam monitoring and irradiation control, delivery‐aware treatment planning, the Monte Carlo‐based BioPhys computational platform integrating physical and biological dose modeling, a hierarchical adaptive workflow, and patient‐specific quality assurance spanning pretreatment, on‐treatment, and post‐treatment verification. This framework provides a technical roadmap for the integrated clinical implementation of CIRT at MCF.

## 1 INTRODUCTION

In Part I of this two‐part review, we summarized the global clinical experience of carbon ion radiotherapy (CIRT), reviewed the evidence supporting its clinical applications, and discussed the anticipated need for CIRT in the United States, together with the clinical and technical demands posed by the anticipated patient population. Building upon that foundation, this article presents the Mayo Clinic Florida (MCF) vision for implementing the first clinical CIRT program in the United States.

While existing CIRT centers in Asia and Europe have established safe and effective CIRT programs, they were designed according to the technological capabilities, clinical priorities, and healthcare environments of their time. Over the past decades, substantial advances have been made in beam delivery, treatment planning, computational modeling, image guidance and adaptive radiotherapy.^1^, ^2^, ^3^, ^4^, ^5^, ^6^, ^7^, ^8^, ^9^ Additionally, the clinical indications for CIRT have continued to evolve as both technological capabilities and clinical evidence have expanded.^10^, ^11^, ^12^

The MCF CIRT program is therefore envisioned as an integrated clinical platform designed to address the anticipated clinical needs and the associated technical demands of CIRT over the coming decades by advancing and integrating developments in accelerator technology, beam delivery, computational modeling, treatment planning, image guidance, and adaptive radiotherapy. The platform is designed as a multi‐ion system capable of delivering both proton and carbon ion therapy within the same treatment session through rapid switching between ion species. The current and future carbon treatment rooms are designed to support this capability while providing a pathway for incorporating additional ion species in the future.

The MCF CIRT program remains under active development, with clinical deployment planned for 2028. Therefore, the technologies and implementations discussed throughout this Vision paper span different stages of maturity. Some underlying methods and concepts, including dose‐driven continuous scanning (DDCS), the MCF MKM radiobiological model, and risk assessment based on failure modes and effects analysis (FMEA), have been investigated in previously published proof‐of‐concept, characterization, or methodological studies conducted as part of the development of the MCF CIRT program.^13^, ^14^, ^15^, ^16^, ^17^, ^18^, ^19^, ^20^ Other components remain under active development, and final operating parameters, quantitative clinical acceptance criteria, and performance benchmarks will be prospectively established and validated during commissioning and clinical workflow development before clinical deployment. This Vision paper describes the design philosophy, engineering translation and integration, and intended clinical implementation of the MCF CIRT program.

## 2 DESIGN PHILOSOPHY

The MCF design philosophy is guided by two overarching principles that address the clinical needs and associated technical demands: (1) fast, accurate, and safe beam delivery and (2) accurate treatment planning and treatment delivery. These principles shape both the technical design of the treatment delivery system and the associated clinical workflow. Additionally, the resulting technical complexity required to achieve these principles necessitates comprehensive, integrated patient‐specific quality assurance (PSQA) throughout the treatment process.

### 2.1 Fast, accurate, and safe beam delivery

A defining feature of the MCF particle therapy delivery system design philosophy is the emphasis on fast, accurate, and safe beam delivery. The projected clinical indications for CIRT in the United States are expected to include a substantial proportion of thoracic and upper abdominal malignancies, making respiratory motion management and real‐time adaptive particle therapy (RTAPT) important components of routine clinical practice. For patients requiring respiratory motion management, fast beam delivery improves the feasibility of breath‐hold treatments, enabling reduced planning margins and thereby improving normal tissue sparing. Fast beam delivery is also an important component of RTAPT because shorter treatment time reduces the likelihood of intrafraction anatomical changes, helping to preserve the dosimetric accuracy of the adapted treatment plan.^21^ More broadly, shorter treatment times reduce the risk of intrafraction motion for all patients.^22^, ^23^, ^24^ Such motion can compromise treatment accuracy by increasing the risk of target underdosage and overdose to organs at risk (OARs), particularly in particle therapy where dose distributions are highly sensitive to motion.^25^

Fast beam delivery also facilitates an efficient clinical workflow, increasing patient throughput, expanding patient access, and enabling more patients to benefit from this advanced treatment modality.

Achieving fast, accurate, safe beam delivery, however, requires a highly integrated beam delivery system capable of delivery treatment efficiently without compromising accuracy or safety. As highlighted in the proton therapy roadmap paper, this requires coordinated advances in beam scanning, energy switching, beam monitoring, and delivery control.^26^ The integration of these capabilities forms the foundation of the MCF treatment delivery system described in section 3.1.

### 2.2 Accurate treatment planning and treatment delivery

Accurate treatment planning extends beyond accurate dose calculation and optimization. It requires that the treatment planning system (TPS) accurately represents how the treatment delivery system (TDS) actually delivers the beam. In current particle therapy practice, however, the TPS and the TDS are largely independent. TPS typically optimizes treatment plans using only simplified delivery parameters, such as spot positions and spot weights, while machine‐specific parameters and control functions, including beam current, beam delivery behavior, and irradiation control logic, remain internal to the TDS. This disconnect affects both dose prediction accuracy and delivery optimization.

Because TPS has limited knowledge of the actual beam delivery process, treatment plans are optimized using a simplified delivery model. For example, current commercial TPSs optimize treatment plans assuming discrete spot scanning (DSS), whereas modern carbon ion facilities employ DDCS, also referred to as raster scanning.^27^ Consequently, the planned dose distribution is inherently based on a delivery model that differs from the actual delivery process, making delivery‐related dose deviations unavoidable.

In addition, because the TPS has limited knowledge of and control over the parameters that govern delivery efficiency, these parameters cannot be optimized on a plan‐specific basis. Instead, to ensure safe and reliable treatment delivery, the TDS employs conservative delivery parameters that are applied uniformly across all treatment plans. Although robust, this strategy inevitably sacrifices delivery efficiency for many individual treatment plans.

To overcome these limitations, next‐generation CIRT requires a delivery‐aware TPS in which the beam delivery process is explicitly modeled. Such a framework enables treatment plans to be optimized not only for the desired dose distribution but also for beam delivery efficiency on a plan‐specific basis. Furthermore, by explicitly incorporating the beam delivery process into treatment planning, it enables accurate prediction of the dose distribution, rather than relying on conservative delivery constraints to compensate for limitations of the TPS delivery model. Though not directly related to the delivery system, the TPS should employ MC‐based dose calculation capable of transporting all relevant ion species, which is critical not only for the accuracy of the physical dose due to nuclear fragmentation, but also for the microdosimetry that serves as input to biological dose algorithms. Robustness to range and patient setup uncertainties is an important consideration in CIRT treatment planning and will be incorporated into the treatment planning process, with specific uncertainty scenarios and settings to be established during commissioning and treatment‐site‐specific clinical protocol development. The delivery‐aware planning framework is described in Section 3.2.

Within the MCF design philosophy, accurate treatment delivery refers to maintaining agreement between the intended and delivered dose distributions throughout the treatment course. Achieving this objective requires accurate patient setup and appropriate management of anatomical changes. CIRT is highly sensitive to inter‐ and intra‐fractional anatomical variations. The anticipated U.S. CIRT patient population is expected to include a large proportion of thoracic, abdominal, and head and neck cancers, many of which are subject to substantial motion or anatomical changes during treatment. Consequently, accurate patient setup and effective and efficient adaptive treatment strategies will be essential to ensure that the delivered dose distribution remains consistent with the intended one throughout the treatment course. The implementation of these capabilities forms the foundation of the MCF patient setup and adaptive treatment framework described in Section 3.3.

### 2.3 Integrated patient‐specific quality assurance

The implementation of the above two overarching design principles introduces substantial technical complexity, placing significantly greater demands on PSQA. Fast beam delivery, complexed delivery control and monitoring systems, delivery‐aware treatment planning, and adaptive clinical workflows collectively introduce levels of technical complexity that require comprehensive PSQA well beyond the scope of conventional PSQA. Historically, PSQA has been treated as a single pretreatment verification task. However, this level of technical complexity requires PSQA to evolve from an isolated pretreatment verification procedure into an integrated QA framework spanning the entire treatment lifecycle. Such a framework should incorporate pretreatment verification, on‐treatment verification, and post‐treatment delivered dose reconstruction to provide comprehensive QA throughout the treatment process. The implementation of this integrated PSQA framework is described in Section 3.4.

## 3 IMPLEMENTATION OF THE DESIGN PHILOSOPHY

### 3.1 Fast, accurate, and safe beam delivery

#### 3.1.1 Accelerator and beam delivery system

The MCF synchrotron supports operation with both proton and carbon ion beams and is designed to accommodate additional ion species in the future. It also enables rapid switching between ion species (∼20 s), making multi‐ion treatment delivery within the same treatment session both feasible and efficient. In addition, the accelerator supports rapid switching between treatment rooms, with switching times of approximately 10 s, enabling efficient operation in a multi‐room clinical environment.

Previous studies have shown that the total beam delivery time (BDT) of synchrotron‐based particle therapy systems is composed of spill change time, energy layer switching time, scan time, spot switching time, and active beam‐on time.^28^ The MCF accelerator was specifically designed to optimize each of these components while maintaining reasonable economic constraints. Spill change time and energy layer switching time were reduced by at least 20% compared with equivalent systems. The scanning system supports high scan speeds, with design values of 250 m/s in y directions for 100 MeV/u carbon beams. Spot switching time is effectively eliminated by DDCS, which maintains continuous beam delivery during spot‐to‐spot transitions instead of turning the beam off between spots, as in conventional DSS.^15^ The active beam‐on time is primarily determined by the beam intensity. The MCF system employs multi‐harmonic RF acceleration and high‐energy injectors, which increase the extracted beam intensity by approximately five‐fold compared to older systems.

Of all BDT components, total spill change time represents the dominant contributor, as a typical spill change requires approximately 2 s, an order of magnitude longer than an energy layer switch.^29^, ^30^ Consequently, minimizing the number of spill changes is critical for achieving fast beam delivery. The MCF particle therapy system addresses this challenge by increasing the maximum charge per spill, extending the extraction time, and substantially improving beam recapture efficiency during multi‐energy extraction (MEE), all of which directly reduce the frequency of spill changes. Preliminary commissioning data demonstrates substantial improvements in these key parameters (Table 1). Compared with other proton therapy systems currently in operation at Mayo Clinic, the maximum charge per spill is increased by approximately five‐fold, the extraction time is doubled, and the MEE recapture efficiency is improved from approximately 50% to more than 95%. In addition, the MCF MEE extraction pattern has been optimized for clinically relevant treatment plans to further minimize the number of required spill changes. Together, these advances are expected substantially reduce BDT.^30^

**TABLE 1:** Key features supporting fast, accurate, and safe beam delivery.

Feature	Description
Multi‐ion operation with rapid species switching	∼ 20 s^a^ ion switch time enables efficient multi‐ion therapy
Fast multi‐room beam switching	∼ 10 s^a^ room switch time improves operational efficiency in a multi‐room treatment facility.
Reduced spill change time	Reduces spill change time to ∼1.6 s^a^ (vs. ∼2 s^b^), shortening overall beam delivery time.
Reduced energy layer switch time in multi‐energy extraction (MEE)	Reduces energy switch time to ∼0.16 s^a^ (vs. ∼0.2 s^b^), shortening overall beam delivery time.
Greater than 95% MEE recapture efficiency	Improves recapture efficiency to > 95%^a^ (vs. ∼ 50%^b^), reducing number of spill changes.
Extended extraction time	Extends the beam extraction window to ≥ 15 s^a^ (vs. 8 s^b^), reducing number of spill changes.
Multi‐harmonic RF acceleration and high energy injector	Increases extracted beam intensity to 40 MU/s^a^ (vs. 8 MU/s^b^) and charge per spill to ∼50 MU^a^, ^c^ (vs. ∼20 MU^b^), enabling higher beam currents and reducing number of spill changes.
High speed scanning	Supports fast beam delivery.
Real‐time synchrotron charge monitoring	Provides real‐time accelerator state information for beam delivery monitoring.
Fast spot position and profile calculation	Enables accurate spot monitoring during ultra‐short spot periods.
Adaptive spot position monitoring (SPM) judgment	Reduces unnecessary beam interruption for very low‐MU spots.
Cumulative skipped SPM judgement tracking	Maintains delivery safety by monitoring accumulated unverified dose.
Dynamic beam‐off break point (BP) generation	Provides adaptive correction of delivery deviations during DDCS.
Fast beam interruption using pulse‐steering (PST) magnet	Beam termination within 20 µs^a^, minimizing unintended dose deposition following beam‐off.

^a^ Quantitative performance values are shown only for representative features where benchmarking is informative, and values are based on preliminary proton beam commissioning at MCF.

^b^ Comparative values refer to the Mayo Clinic proton therapy system currently in clinical operation, commissioned in 2016.

^c^ MU is calibrated such that 200 MU delivers a uniform 2 Gy dose to a 1‐L cubic target with the isocenter positioned at a depth of 15 cm in water. Under this calibration, 1 MU corresponds to approximately (0.4–1.0)×10^9^ protons, or (2–5)×10^7^ carbon ions.

These accelerator and beam delivery innovations establish the foundation for fast beam delivery. Achieving this performance accurately and safely requires sophisticated beam delivery monitoring and control system described in the following subsection.

#### 3.1.2 Beam delivery monitoring and control system

The MCF particle therapy system employs DDCS delivery approach designed to reduce BDT and improve treatment efficiency.^14^, ^15^ Unlike conventional DSS, in which the beam is off between every spot transition and the prescribed MU are delivered only at stationary spot positions, DDCS delivers dose continuously while the scanning magnets move between spots. Although this approach reduces beam‐off time,^14^, ^15^ it also introduces new challenges for delivery monitoring and control. During spot‐to‐spot transitions, dose is deposited continuously along the scan path (move dose). The magnitude and spatial distribution of the move dose depend on the scan time, beam current, and scan trajectory.^14^, ^16^, ^31^ Consequently, fluctuations in beam delivery can alter both the magnitude and spatial distribution of the move dose, necessitating a more sophisticated beam monitoring and delivery control strategy.

To date, most clinical DDCS implementations have adopted conservative approaches to minimize the dosimetric impact of move dose. These strategies typically include reducing beam current so that the majority of the dose is delivered at stationary spot positions and introducing beam‐off control points (breakpoints, BPs) to eliminate move dose during long spot‐to‐spot transitions or transitions expected to produce excessive move dose.^17^ Although effective in reducing move dose related uncertainty, both strategies increase BDT and reduce delivery efficiency.

The MCF system was designed around a fundamentally different philosophy. Rather than preserving delivery accuracy through conservative delivery constraints that degrade delivery efficiency, the goal is to enable high‐speed DDCS delivery while maintaining accurate and safe beam delivery through three complementary components: (1) a sophisticated beam delivery monitoring and control system, (2) a delivery‐aware treatment planning that explicitly models the relevant beam delivery behaviors, and (3) a comprehensive PSQA program. The beam delivery monitoring and control system is described in this section, while the delivery‐aware treatment planning framework and integrated PSQA program are presented in the sections of 3.2 and 3.4.

To support this philosophy, the MCF beam delivery monitoring and control system integrates four complementary functions: continuous accelerator monitoring, high speed spot monitoring, adaptive irradiation control, and rapid beam interruption. Continuous accelerator monitoring is achieved through a dedicated synchrotron charge monitoring system based on direct current transformer (DCCT) measurements, which continuously tracks the circulating beam charge throughout beam extraction and provides real‐time information on the instantaneous accelerator state. High speed spot monitoring is enabled through fast spot position monitor (SPM) calculation capable of accurately determining spot position and profile using very small monitoring signals (2×10^4^ protons or 1×10^3^ carbon ions) within extremely short spot periods (30 µs). Building upon these monitoring capabilities, the irradiation control system incorporates adaptive SPM judgment logic that selectively bypasses SPM evaluation for low‐MU spots to avoid unnecessary beam interruptions associated with the negligible dosimetric impact due to variations associated with these spots. To ensure delivery safety, the system continuously tracks the cumulative unverified dose and automatically initiates corrective actions, including on‐the‐fly BP generation or beam interruption, before the accumulated unverified dose exceeds predefined safety tolerances.^13^ The use of a fast pulse‐steering (PST) magnet enables beam interruption within 20 µs, minimizing the delay between the beam‐off command and actual beam termination, reducing unintended dose deposition during high beam current delivery.

The key features of the accelerator, beam delivery, and irradiation control system are summarized in Table 1.

#### 3.1.3 Failure mode analysis and risk‐informed commissioning

The implementation of the MCF fast, accurate, and safe beam delivery strategy introduces a novel beam delivery paradigm that extends beyond the scope of existing commissioning and quality assurance guidelines and creates potential failure modes that have not been previously evaluated. To support the safe clinical implementation of this approach, a prospective FMEA was performed to systematically identify, quantify, and mitigate risks associated with the clinical implementation of this beam delivery system.^13^

Following the AAPM TG‐100 framework,^32^a detailed process map of the MCF beam delivery monitoring and control system was developed, and 29 potential failure modes were identified. The analysis identified several failure modes associated with high‐speed DDCS delivery and the novel beam delivery control strategies described above, including scan‐path‐ and scan‐time‐dependent move dose deposition during beam transit, flap dose generated between beam‐off command execution and actual beam termination at high beam current delivery, dose monitor performance under high beam current and small spot size conditions, and failures associated with novel adaptive irradiation control and dynamic beam‐off BP generation.^13^

The primary outcome of the FMEA was the development of a risk‐informed commissioning framework tailored to the MCF particle therapy system.^13^ The framework includes verification of dose monitor performance under extreme operating conditions, validation of adaptive SPM judgment logic, and testing of the delivery control system under clinically relevant extreme delivery scenarios. The FMEA also identified several delivery‐related effects that require explicit modeling within the treatment planning process, including scan path and scan time characterization and modeling of move dose and flap dose. The delivery‐aware treatment planning framework developed to address these requirements is described in the following section.

### 3.2 Accurate treatment planning

#### 3.2.1 Delivery‐aware treatment planning framework

To fully realize the benefits of the MCF beam delivery system and achieve accurate treatment planning, a delivery‐aware treatment planning framework was developed that explicitly models machine‐specific beam delivery characteristics and enables accurate prediction of the delivered dose distribution. The framework incorporates scan path and scan time modeling to predict the location and magnitude of transit dose deposition (move MU), flap dose modeling to account for unintended dose delivered during beam termination latency, and realistic simulation of spot delivery sequence and timing based on the behavior of the irradiation control system. It further incorporates a plan deliverability assessment module that uses beam current fluctuation models derived from machine measurements to simulate realistic beam delivery behavior and identify treatment plans that are likely to trigger delivery control interventions or treatment interruptions.

The development of this delivery‐aware treatment planning framework required comprehensive characterization of machine‐specific beam delivery behaviors. To support this effort, an extensive research program was established through collaborations with existing particle therapy centers employing the same vendor delivery system as ours. Although the MCF system incorporates substantially enhanced performance capabilities and operating parameters, it is based on the same fundamental beam delivery architecture used by other centers employing the same vendor's technology. Consequently, generalized mathematical models describing beam delivery behavior can be developed before MCF commissioning and subsequently refined using site‐specific commissioning data. Experimental measurements were performed to characterize beam current ramp‐up behavior, extracted beam current fluctuations, scanning magnet trajectories, flap dose, and other fundamental beam delivery characteristics.^33^, ^34^, ^35^, ^36^, ^37^ In parallel, a series of in silico treatment control system simulations were conducted to evaluate the impact of beam delivery behaviors.^14^, ^15^, ^16^, ^17^, ^18^, ^31^, ^38^ These studies guided the development of the delivery‐aware treatment planning framework by identifying which beam delivery behaviors required explicit modeling and the degree of modeling accuracy necessary for accurate dose prediction.

Once the MCF system becomes operational, machine‐specific parameters will be measured during commissioning and incorporated into the previously developed mathematical models of beam delivery behavior. Because the modeling framework has already been established, commissioning will primarily involve parameterizing the model using MCF‐specific measurements, substantially accelerating the commissioning process.

#### 3.2.2 BioPhys computational platform

The MCF BioPhys platform is an in‐house MC‐based computational framework currently under development to support particle therapy dose calculation, biological dose assessment, treatment delivery simulation, and computational PSQA. Built upon the Geant4‐based TOPAS toolkit,^39^, ^40^, ^41^ BioPhys is designed to extend beyond a conventional MC dose engine by integrating particle transport physics, radiobiological modeling, machine‐specific delivery simulation, and delivered dose reconstruction within a unified computational framework. The delivery‐aware treatment planning capabilities described in the previous section are designed to be implemented within this platform, providing a common computational environment for independent dose calculation, delivery simulation, and delivered dose reconstruction. Figure 1 illustrates the envisioned architecture of the BioPhys computational platform.

**FIGURE 1:**
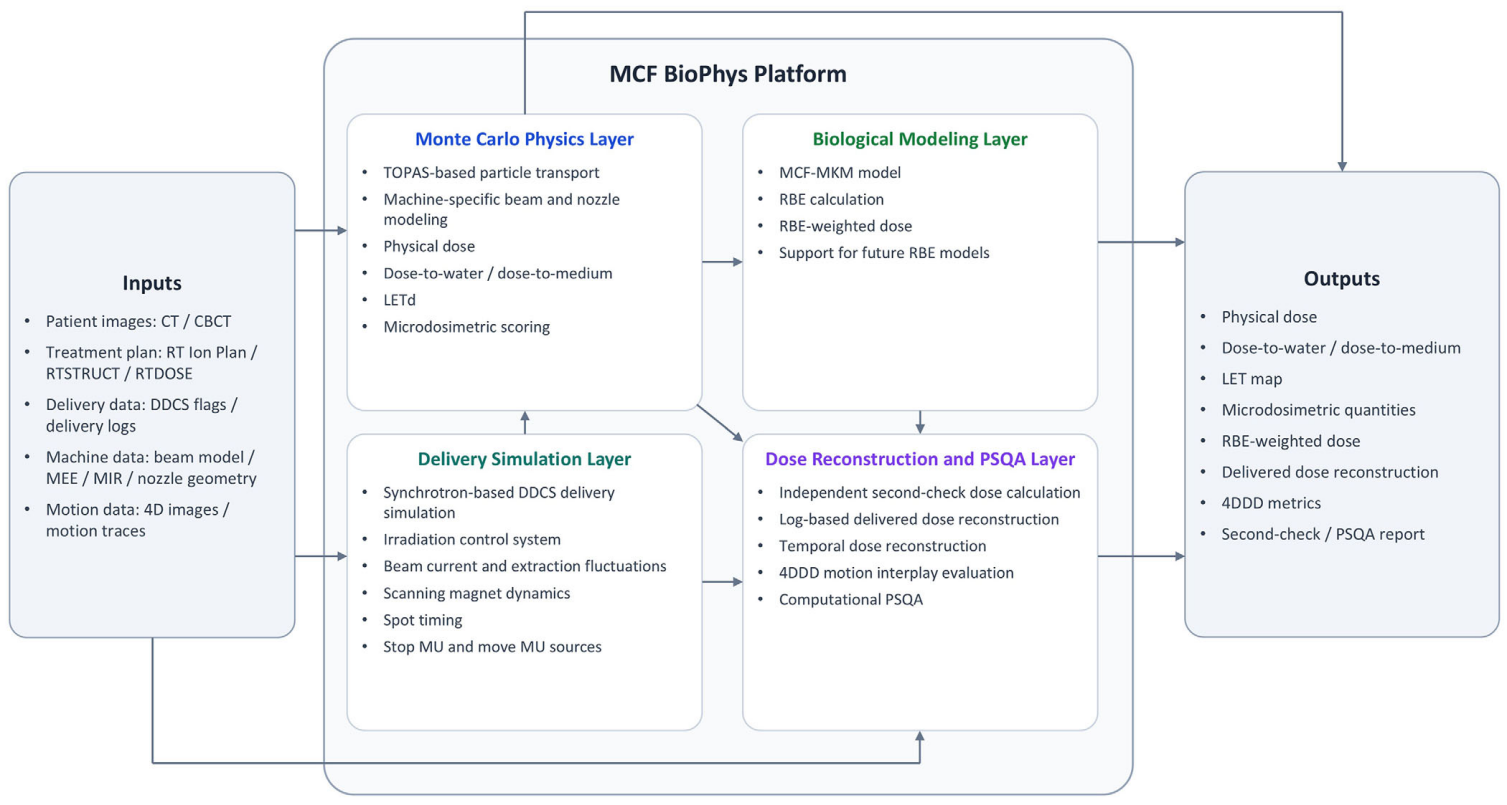
Envisioned architecture drawing for the biophysic dose calculation platform.

A distinguishing feature of BioPhys is its comprehensive representation of the treatment delivery system. Rather than modeling only particle transport, the platform is designed to incorporate machine operation parameters together with detailed representations of the irradiation control system and delivery logic. The platform is designed to explicitly simulate the synchrotron‐based DDCS delivery process, including beam current ramp‐up, extraction fluctuations, scanning magnet dynamics, scan‐path trajectories, and spot‐by‐spot delivery timing. To accurately model DDCS dose delivery, including both the dose deposited at planned spot locations (stop MU) and the dose delivered during spot‐to‐spot transitions (move MU), the beam trajectory is discretized into multiple move sources, each assigned a position, beam intensity, and MU based on commissioned machine characteristics. This approach enables accurate simulation of move dose and realistic reconstruction of the delivered dose distribution.

At the physics layer, BioPhys performs detailed MC simulations for particle therapy and is designed to be expandable to support all ion species. The platform can reconstruct voxel‐by‐voxel physical dose distributions, dose‐averaged linear energy transfer (LETd), microdosimetric quantities including lineal energy spectra required for biological dose modeling. Microdosimetric spectra are generated using precomputed MC lookup libraries,^42^ providing an efficient representation of microscopic energy deposition while preserving the accuracy of detailed particle transport simulations.

Building upon these physics quantities, the biological modeling layer reconstructs voxel‐level RBE‐weighted dose using microdosimetric‐based radiobiological models. The framework currently implements the MCF Microdosimetric Kinetic Model (MCF 53‐60MKM)^19^, ^20^ while maintaining the flexibility to incorporate additional or future RBE models without modifying the underlying particle transport calculations. This modular architecture is designed to enables retrospective biological dose reconstruction using newly developed RBE models, facilitating continuous refinement of biological dose estimation and future dose–response investigations.

Because the physical, biological, and delivery models are integrated within a common computational framework, BioPhys is designed to support temporal dose reconstruction and four‐dimensional dynamic dose (4DDD) evaluation. By combining delivery timing with patient motion information, the platform can enable quantitative assessment of motion interplay effects. Furthermore, by integrating time‐resolved delivery log data with patient imaging acquired throughout treatment, BioPhys can enable reconstruction of the delivered physical and biological dose on the patient's anatomy at the time of treatment.

### 3.3 Accurate treatment delivery

To achieve accurate treatment delivery, particularly for the anticipated U.S. CIRT patient population characterized by complex disease sites susceptible to anatomical variation, an effective and efficient clinical framework for patient setup and adaptive treatment is essential. These clinical challenges are further amplified in CIRT because of the sharp dose gradients of carbon ion beams and their sensitivity to setup uncertainties and anatomical changes. Current clinical practice relies on image‐guided radiotherapy (IGRT), which aligns the daily patient anatomy with the planning CT. IGRT assumes that accurate geometric alignment is an appropriate surrogate for accurate intended dose distribution. However, for treatment sites characterized by large target volumes such as head and neck or substantial tissue deformation such as breast, a single rigid transformation cannot simultaneously align the entire irradiation volume. Consequently, the optimal registration may not correspond to the patient position that best reproduces the planned dose distribution. This is especially true for particle therapy where the distributions are highly sensitive to tissue density variations and anatomical changes along the beam path.

Cheung et al. proposed dose‐guided patient positioning (DGPP), which replaces anatomy‐based setup by optimizing patient positioning to better reproduce the planned dose distribution.^43^This work and subsequent studies in proton therapy demonstrated DGPP more effectively achieved the planned dose distribution than conventional anatomy‐based positioning.^43^, ^44^ However, the effectiveness of DGPP is inherently limited to anatomical changes that can be corrected through rigid patient repositioning. When anatomical deformation exceeds this corrective capability, adaptive replanning becomes necessary.^44^

RTAPT re‐optimizes the treatment plan on the anatomy of the day immediately before treatment delivery. Significant advances in daily imaging, deformable image registration, automatic contour propagation, rapid dose calculation, fast treatment plan optimization, and automated QA have brought RTAPT closer to routine clinical implementation.^45^, ^46^, ^47^ More recently, the first clinical implementation of RTAPT workflow was reported.^9^ Despite these advances, RTAPT workflows remain resource intensive, requiring substantial computational resources, extensive workflow automation, and multidisciplinary clinical coordination, while also resulting in prolonged patient on‐table times that limit widespread clinical adoption. These limitations motivate a more efficient adaptive treatment strategy.

The MCF CIRT program proposes a hierarchical patient setup and adaptive workflow (Figure 2). The workflow integrates IGRT, DGPP, and RTAPT into a unified, dosimetry‐driven clinical workflow. Unlike most reported RTAPT workflows, which proceed directly from image‐guided positioning to real‐time adaptive replanning, the proposed MCF workflow incorporates DGPP as an intermediate decision layer. Previous studies have demonstrated that DGPP can improve restoration of the planned dose distribution compared with image‐guided positioning, providing an opportunity to recover clinically acceptable dosimetry without treatment plan adaptation.^43^, ^44^ More recently, Oosterhof et al. developed an automated DGPP workflow using fast MC dose reconstruction and demonstrated clinically acceptable optimization times (149 ± 51 s) together with improved target coverage compared with anatomy‐based positioning in head and neck proton therapy, providing further evidence that DGPP can be implemented at a clinically relevant timescale.^48^ By incorporating DGPP as an intermediate step, the proposed workflow establishes a hierarchical decision‐making strategy in which progressively more resource‐intensive interventions are selected according to dosimetric need. Optimized rigid repositioning may first be used to restore the intended dose distribution, while real‐time adaptive replanning is reserved for cases in which acceptable dosimetry cannot be recovered through patient repositioning alone. Although the clinical decision process is hierarchical, implementation of the IGRT, DGPP, and RTAPT pathways is designed to proceed in parallel following acquisition of the daily volumetric image to minimize delays associated with sequential processing. Because biological dose is the driving factor for treatment evaluation and adaptation, the proposed framework uses MC‐based dose calculation to accurately model the microdosimetric quantities required for biological dose calculation.

**FIGURE 2:**
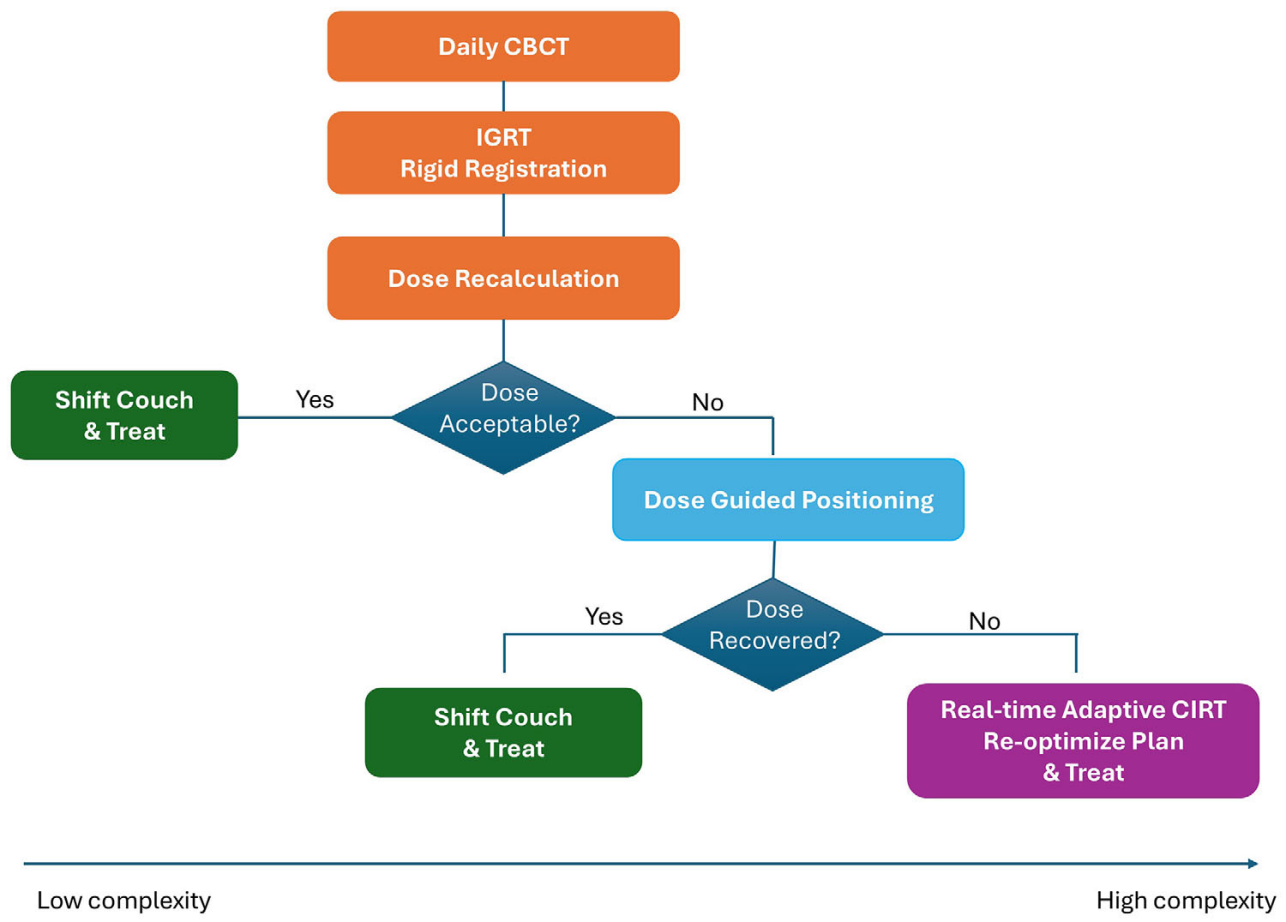
Proposed MCF biological dose driven hierarchical decision workflow. Although clinical decisions are hierarchical, the computational processes supporting IGRT, DGPP, and RTAPT are designed to execute in parallel. The dose acceptability criteria will be prospectively established and validated before clinical deployment as part of treatment‐site‐ and protocol‐specific commissioning and clinical workflow development. These criteria are expected to incorporate target coverage metrics and OAR dose constraints defined by clinical protocols.

A distinguishing feature of the MCF RTAPT framework is that the entire adaptive treatment workflow is implemented within the local clinical treatment network. To support the stringent timing requirements of RTAPT, all time critical functions, including image processing, dose reconstruction, treatment evaluation, and adaptive replanning, are performed locally within this network and, where possible, in parallel. By avoiding repeated transfer of large imaging datasets between enterprise information systems and the treatment delivery environment, the proposed architecture minimizes communication latency and enables efficient integration of adaptive planning into the routine clinical workflow. Following clinical approval, the adapted RT ion plan can be transferred directly to the treatment delivery system through dedicated vendor‐supported interfaces, supporting practical real‐time clinical implementation. The local network and parallel computing architecture represents a design strategy intended to minimize computational and communication latency. Because the MCF CIRT RTAPT platform remains under development, quantitative timing benchmarks for these time‐critical processes are not yet available. The computational time budget for individual components and the overall adaptive workflow will be prospectively characterized and validated as platform development is completed and during subsequent system commissioning and clinical workflow development before clinical deployment.

### 3.4 Integrated patient‐specific quality assurance

Built upon the BioPhys computational platform, the MCF CIRT program is developing a comprehensive PSQA framework spans the entire treatment process. Rather than treating PSQA as a single pretreatment verification procedure, the proposed framework is designed to extend PSQA to the pretreatment, on‐treatment, and post‐treatment stages.

#### 3.4.1 Pretreatment independent dose calculation

Although conventional measurement‐based PSQA has long served as the clinical standard for treatment plan verification,^49^ its reliance on homogeneous phantom measurements provides only a surrogate assessment of dose in patient anatomy and has inherent limitations in evaluating treatment planning accuracy and delivered dose in patient anatomy.^50^, ^51^, ^52^ These limitations have driven the development of computational PSQA approaches based on independent dose calculation (IDC) and treatment log‐file analysis, which have been shown to improve sensitivity to treatment planning and delivery errors, and also reduce clinical workload while maintaining a high level of patient safety.^1^, ^3^, ^50^, ^53^, ^54^, ^55^, ^56^, ^57^, ^58^, ^59^, ^60^ Recent PTCOG recommendations similarly recognize independent dose calculation and log file‐based verification as important complementary components of modern computational PSQA.^61^

Building upon this established workflow, the proposed MCF framework extends pretreatment IDC beyond conventional verification in two important directions: delivery‐aware and biologically informed treatment assessment. Leveraging the spot‐by‐spot treatment delivery simulation capability of the BioPhys platform, patient‐specific respiratory motion derived from 4DCT can be combined with the simulated temporal spot delivery sequence to perform 4DDD simulation before treatment delivery, enabling prospective evaluation of patient‐specific motion interplay and treatment robustness. In addition, by integrating multiple clinically adopted and newly developed RBE models within the BioPhys platform, treatment plans can be evaluated under different biological assumptions. This capability is particularly important for CIRT, where the lack of a universally accepted clinical RBE model remains a well‐recognized challenge, allowing biological uncertainty to be incorporated into pretreatment PSQA and providing a more comprehensive assessment of treatment plan robustness and clinical acceptability.

#### 3.4.2 On‐treatment real‐time PSQA

Machine control (steering) files for PSQA was proposed by Meier et al. ^59^ and subsequently evaluated by Matter et al. ^50^ as an alternative to conventional measurement‐based PSQA, demonstrating improved sensitivity for detecting data transfer errors and workflow failures compared with phantom measurements. Despite these promising results, machine control file verification has not been widely incorporated into routine clinical workflows. One major barrier to broader clinical implementation is that the translated machine control files are generally not accessible outside the treatment delivery system (TDS), and their proprietary format limits independent interpretation by external QA software.

Within the proposed MCF real‐time PSQA (RTPSQA) framework, access to translated treatment delivery data is provided through a dedicated interface developed in collaboration with the particle therapy system vendor, allowing our RTPSQA platform to obtain both the translated machine‐format treatment file and the corresponding translation rules used by the treatment control system (TCS) (Figure 3). This framework provides independent verification immediately before beam delivery to assess both the integrity of treatment plan transfer and the accuracy of translation from the physician‐approved DICOM RT Ion Plan into machine‐specific control instructions. The RTPSQA platform obtains the translated machine‐format treatment file together with the corresponding translation rules from the TCS, reconstructs a DICOM‐equivalent representation, and performs parameter‐by‐parameter comparison with the approved DICOM treatment plan. This independent verification can provide end‐to‐end assessment of the digital treatment delivery chain and identify discrepancies before patient irradiation.

**FIGURE 3:**
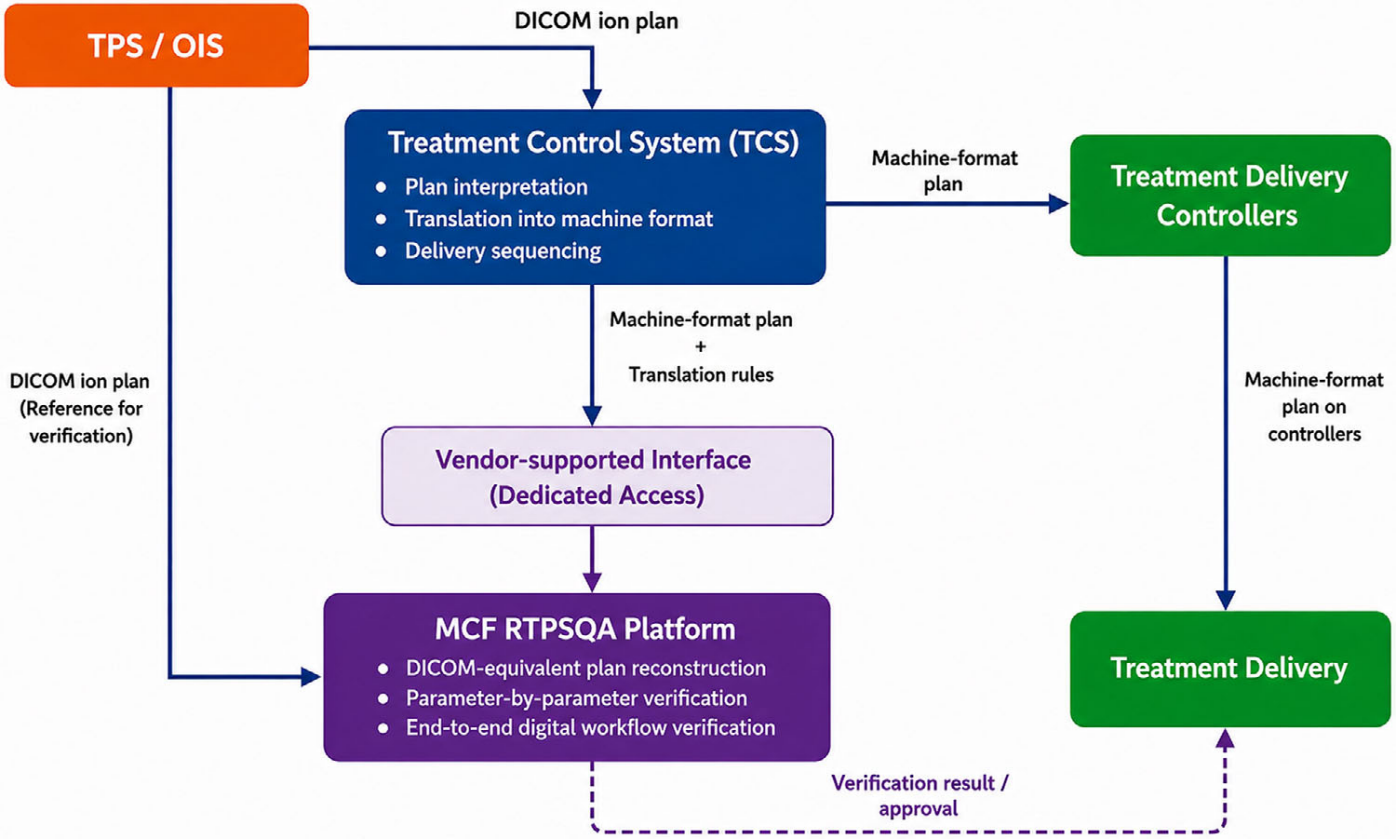
System architecture of the real‐time patient‐specific quality assurance (RTPSQA) framework for independent verification of the digital treatment delivery chain. The physician‐approved DICOM ion plan is translated by the treatment control system (TCS) into machine‐specific control instructions for treatment delivery. The RTPSQA server independently retrieves the translated machine‐format treatment file and the corresponding translation rules from the TCS database, reconstructs a DICOM‐equivalent representation of the treatment plan, and performs a parameter‐by‐parameter comparison with the original DICOM plan immediately before beam delivery.

#### 3.4.3 Post‐treatment delivery log‐based PSQA

Treatment delivery log files have become an increasingly important component of computational PSQA.^50^, ^55^, ^59^, ^62^, ^63^, ^64^, ^65^ The proposed MCF framework extends conventional post‐treatment log‐based PSQA by enabling biological dose evaluation using multiple RBE models. Depending on the clinical objective, dose reconstruction may be performed on the original planning CT to evaluate delivery errors alone or on synthetic CT images generated from daily patient setup images to assess the combined effects of delivery uncertainty, patient setup variation, and anatomical changes. The reconstructed dose distributions may also be evaluated by using different RBE models to assess the impact of biological dose uncertainty. When appropriate, reconstructed dose distributions from individual treatment fractions may be accumulated throughout the treatment course to evaluate treatment consistency over time and provide quantitative information to support adaptive treatment decisions when clinically meaningful deviations are observed.

These pretreatment, on‐treatment, and post‐treatment verification components establish the proposed integrated PSQA framework, providing patient‐specific verification throughout the treatment process to safely support the technical complexity required to achieve the MCF design philosophy.

## 4 CONCLUSION AND FUTURE PERSPECTIVES

The anticipated future CIRT patient population, characterized by a predominance of clinically complex disease sites, place significant demands on treatment delivery and treatment management. These demands form the foundation of the MCF design philosophy, which emphasize fast, accurate, and safe beam delivery, delivery‐aware treatment planning, adaptive treatment, and PSQA through the entire treatment process as complementary components of a unified clinical platform.

The proposed MCF program is intentionally designed as an evolving clinical platform designed to incorporate continued advances in accelerator technology, treatment planning, adaptive treatment, and radiobiological modeling. As clinical experience grows and new technologies emerge, continued refinement of these integrated components will be essential for maximizing the long‐term clinical impact of CIRT in the United States.

## AUTHOR CONTRIBUTIONS

Conceptualization, Xiaoying Liang and Chris J Beltran; Writing‐original draft, Xiaoying Liang; Writing‐review & editing, Chris J Beltran and Xiaoying Liang; Both authors have read and approved the final manuscript.

## CONFLICT OF INTEREST STATEMENT

The authors declare no conflicts of interest.

## Data Availability

The data that supports the findings of this study are available from the corresponding author upon reasonable request.
